# Infection prevention in general medical wards: A call to action

**DOI:** 10.7196/AJTCCM.2019.v25i4.036

**Published:** 2019-12-06

**Authors:** S Maasdorp, S Potgieter, R Lodewyk

**Affiliations:** 1 Division of Pulmonology and Critical Care, Department of Internal Medicine, Faculty of Health Sciences, University of the Free State, Bloemfontein, South Africa; 2 Division of Infectious Diseases, Department of Internal Medicine, Faculty of Health Sciences, University of the Free State, Bloemfontein, South Africa; 3 Infection Prevention and Control, Free State Department of Health, Bloemfontein, South Africa

## Editorial

Healthcare-associated infection (HCAI) is defined as ‘an infection acquired in hospital by a patient who was admitted for a reason other than that infection. This includes infections acquired in the hospital but appearing after discharge, and also occupational infections among staff of the facility’.^[1]^ The prevalence of HCAI in high-income countries is 7.6%, but can be as high as 19.1% in low-income countries.^[2]^ The risk of developing HCAI in the intensive care unit is even higher, with an estimated 30% of patients affected in high-income settings v. up to 88.9% of patients in low-income settings.^[2]^ Much more needs to be done to reduce the prevalence of HCAI.

Tuberculosis (TB) is one of the top 10 causes of death globally and remains the leading cause of death by a single infectious agent.^[3]^ It is estimated that 10 million people worldwide developed symptomatic TB infection in 2017, with 2.48 million of these new infections occurring in Africa.^[3]^ In 2016, TB, influenza, pneumonia and chronic lower respiratory disease were among the top 10 leading causes of death in South Africa (SA).^[4]^ In a setting of high TB prevalence, *Mycobacterium tuberculosis* is also a leading cause of community-acquired pneumonia.^[5]^ Lack of respiratory isolation facilities in hospitals in low/middle-income countries can result in patients with both diagnosed and undiagnosed TB disease being admitted to general medical wards for treatment.^[6]^ If unsuspected TB cases are admitted to general wards, the risk of transmitting TB to patients with silicosis, diabetes mellitus, chronic renal failure and those undergoing treatment with corticosteroid therapy is significantly increased.^[7]^ Even among healthcare workers, a systematic review found that the prevalence of latent TB was on average 54%, and the annual incidence of TB disease ranged between 69 and 5 780 per 100 000.^[8]^ Tuberculosis is therefore also an important occupational disease among healthcare workers.^[9]^

In 2016, the WHO published guidelines on infection prevention and control (IPC) programmes that can easily be adapted for implementation in general medical wards of low-resource institutions.^[10]^ The guidelines recommend that an IPC coordinator ideally be delegated to ensure enforcement and evaluation of infection control policies. Risk assessment to promptly identify suspected TB patients and to separate them as quickly as possible is critically important.^[11]^ This may be hampered by the lack of isolation facilities in low-resource environments.^[6]^ However, the availability of high-quality molecular diagnostic tests such as the Xpert MTB/ rif improves the sensitivity of screening algorithms and allows a rapid diagnosis, separation of the confirmed positive patient and therefore optimal use of limited isolation facilities.^[11]^ Transfer of respiratory pathogens can occur by either direct or indirect contact transmission, droplet transmission or airborne transmission.^[12]^ It is advisable that patients with suspected or confirmed pulmonary TB be isolated in a single room with negative pressure and en suite facilities.^[13]^ This is, however, not always possible. Where negative pressure facilities are not available, natural ventilation can be an effective and cost-efficient way of ensuring adequate air mixing.^[14]^ Alternative environmental control strategies include extractor fans installed to the outside away from the intake, ultraviolet lights that inactivate airborne droplet nuclei, skylights and high-efficiency particulate (HEPA) filters.^[15]^

Concerns have been raised about the potential for cross-infection among patients with bronchiectasis visiting out-patient clinics or who are admitted to hospital wards.^[16]^ Aerosols produced by coughing can spread viable *Pseudomonas aeruginosa* up to 4 metres and can remain suspended in the air for at least 45 minutes.^[17]^ One of the cardinal features of bronchiectasis is that the abnormal airways can become colonised with pathogenic bacteria.^[18]^ *P. aeruginosa*, *Staphylococcus aureus* and *Burkholderia cepacia* colonisation portends a worse prognosis, and a mainstay of treatment is prevention and eradication of these pathogens at the time of first recognition.^[19]^ Previous studies reported environmental or person-to-person transmission of *B. cepacia* and *P. aeruginosa* among patients with cystic fibrosis.^[20]^ This led to a practice of cohorting patients by colonising pathogen and preventing direct contact between patients.^[21]^ Furthermore, a common occurrence is for sputum samples to be requested from patients while they are in a hospital room among other patients. This high-risk practice can be easily addressed at low cost by providing inexpensive sputum collection booths in hospital wards.^[22]^

Apart from airborne transmission, HCAI can also occur from colonisation of endogenous body sites such as the skin, nose and gastrointestinal tract, often from contact with the hands of healthcare providers or medical equipment.^[2]^ Environmental cleaning is therefore undoubtedly an important aspect of preventing the spread of hospital-acquired pathogens.^[23,24]^ Cleaning services in hospitals are often contracted out to third parties who invariably use detergents and water to ensure aesthetically clean floors and bathrooms. The risk of spreading pathogenic organisms is, however, higher from surfaces in close proximity to the patients, such as bed rails, bedside lockers, infusion pumps, nurse call button and door handles.^[23]^ Hand-touch sites such as computer keyboards have also been implicated in harbouring pathogens.^[23]^ It is therefore important to emphasise environmental cleaning, especially concentrating on using disinfectants rather than only detergents on surfaces that are common hand-touch sites. Hand hygiene facilities should also be available at the point of care.^[10]^ In their guidelines for preventing transmission of *M. tuberculosis* in healthcare settings, the Centers for Disease Control and Prevention advocate a number of simple, cost-effective strategies that can be implemented in resource-limited settings to prevent nosocomial transmission of respiratory pathogens. These include the provision of an alcohol hand rub bottle at the bedside and the encouragement of hand hygiene after coughing. Patients should also be educated in terms of cough etiquette. A continuous supply of appropriate personal protective equipment for staff members who come in contact with patients with airborne infectious diseases is imperative. The door of the room opening to passages must be kept closed and no fans should be used in rooms where airborne risk infectious patients are placed. This can only increase the spread of infectious particles to the rest of the ward. Surgical masks should be provided to patients who are coughing productively, and this should also be remembered when these patients are transferred within the hospital. Where en suite bathroom facilities are not available, infectious patients should wear surgical masks when visiting ablution facilities that are shared with other patients.^[25]^

## Conclusion

Much can be said about infection prevention and control practices (or lack thereof) at public sector healthcare facilities in SA. Only with dedication from hospital management, joining hands with infection, prevention and control practitioners, and acting on the concerns of healthcare workers and patients, can the surge of healthcare-associated infections be halted.
